# Isolation of Yeast Strains with Higher Proline Uptake and Their Applications to Beer Fermentation

**DOI:** 10.3390/jof9121137

**Published:** 2023-11-24

**Authors:** Ryoya Tanahashi, Akira Nishimura, Minh Nguyen, Irnayuli Sitepu, Glen Fox, Kyria Boundy-Mills, Hiroshi Takagi

**Affiliations:** 1 Institute for Research Initiatives, Nara Institute of Science and Technology, 8916-5 Takayama-cho, Ikoma 630-0192, Nara, Japan; tanahashi.ryoya.ti3@bs.naist.jp; 2 Department of Food Science and Technology, University of California Davis, One Shields Ave, Davis, CA 95616, USA; iiinguyen@ucdavis.edu (M.N.); irsitepu@ucdavis.edu (I.S.); gpfox@ucdavis.edu (G.F.); klbmills@ucdavis.edu (K.B.-M.); 3 Division of Biological Science, Graduate School of Science and Technology, Nara Institute of Science and Technology, 8916-5 Takayama-cho, Ikoma 630-0192, Nara, Japan

**Keywords:** *Saccharomyces cerevisiae*, non-*Saccharomyces*, proline utilization, yeast culture collection, beer fermentation

## Abstract

Although proline is the most or second most abundant amino acid in wort and grape must, it is not fully consumed by the yeast *Saccharomyces cerevisiae* during alcoholic fermentation, unlike other amino acids. Our previous studies showed that arginine, the third most abundant amino acid in wort, inhibits the utilization of proline in most strains of *S. cerevisiae*. Furthermore, we found that some non-*Saccharomyces* yeasts utilized proline in a specific artificial medium with arginine and proline as the only nitrogen source, but these yeasts were not suitable for beer fermentation due to their low alcohol productivity. For yeasts to be useful for brewing, they need to utilize proline and produce alcohol during fermentation. In this study, 11 *S. cerevisiae* strains and 10 non-*Saccharomyces* yeast strains in the Phaff Yeast Culture Collection were identified that utilize proline effectively. Notably, two of these *S. cerevisiae strains*, UCDFST 40-144 and 68-44, utilize proline and produce sufficient alcohol in the beer fermentation model used. These strains have the potential to create distinctive beer products that are specifically alcoholic but with a reduction in proline in the finished beer.

## 1. Introduction

*Saccharomyces cerevisiae* plays an important role in alcoholic fermentation, brewing a rich variety of alcoholic beverages, such as wine, spirits, and beer. Apart from producing high concentrations of alcohol, yeast also plays a crucial role in determining the aroma and taste of the final product. Therefore, the quality of alcoholic beverages is highly dependent on the metabolic characteristics of yeast. The availability of nutrients, particularly nitrogen, is a crucial factor in yeast growth, production of aroma compounds, and fermentation rate.

The main requirement in beer fermentation is wort, which contains a wide range of nitrogen-containing compounds. Beer yeast can assimilate free amino acids and ammonium ions as nitrogen sources. Yeast can also utilize di- and tri-peptides, but with lower efficiency [1]. Free amino acids are the main nitrogen sources available to yeast. The composition of free amino acids in wort has been well studied, and proline is one of the abundant amino acids [2]. Procopio et al. showed that proline assimilation has an impact on the synthesis of aroma-active compounds [3]. Flor yeasts, which can grow as a film on the wine surface and are responsible for the biological aging of Sherry wine, are likely to consume proline during the wine aging process due to being under aerobic conditions on the surface [4]. This process may contribute to the unique flavor of Shery wine. Moreover, we reported that proline catabolism regulates the longevity of *S. cerevisiae* cells [5]. Unlike other amino acids, however, yeasts do not fully consume proline during alcoholic fermentation, which leads to high proline concentrations in beer and related products [6,7,8]. Excess proline remaining in fermented products generally has the negative effect of bitterness and low-acid taste, and binding of proline to polyphenols can result in unwanted hazes [9,10,11]. Hence, to improve the quality of beer and other fermented products, it would be beneficial to have yeast strains that can fully utilize proline.

Previously, Omura et al. attempted to reduce proline levels by mutating the proline transporter Put4 [12]. They constructed a Put4 variant in which all nine N-terminal lysine residues were replaced by arginine. This Put4 variant was insensitive to downregulation via ubiquitination and endocytosis. This approach promoted proline uptake and successfully reduced proline levels in the medium. However, the strains developed in this study were not practically applied because foods and beverages made from genetically modified organisms (GMOs) are not always acceptable to the public [13]. Conventional practical methods for generating superior yeast mutants involved self-cloning or isolating desirable yeasts from the environment. Both approaches are known to be time-consuming and require much effort. More recently, culture collections have provided researchers with a time-saving and more effective way to identify potential desirable candidates. The Phaff Yeast Culture Collection (https://phaffcollection.ucdavis.edu) is the world’s fourth-largest public collection of wild yeasts. Its strains are sourced from the natural environment, including agriculture, food, and fermented food processing. Importantly, the Phaff collection provides researchers with a broad diversity of wild strains that have not been genetically modified. The Phaff collection is often used for both basic and applied research, including the production of food ingredients, biofuels, fine and bulk chemicals [14]. Thus, the Phaff collection has great potential in industrial applications.

We previously found that arginine, the third most abundant amino acid in wort, inhibits proline utilization in *S. cerevisiae* [15,16]. To study proline uptake in non-Saccharomyces yeast species, we screened yeasts from the Phaff collection for their ability to utilize proline using an artificial medium with arginine and proline as the only nitrogen source. Several yeast genera including Zygoascus, Galactomyces, and Magnusiomyces, were able to utilize proline. However, they were not suitable for beer fermentation due to their low alcohol productivity in wort. For practical use in wort fermentation, yeasts must be able to utilize proline and produce alcohol.

In this study, we examined the applicability of the isatin method for the rapid estimation of proline levels in a nitrogen-rich medium such as a synthetic complete medium. Furthermore, we identified 11 *S. cerevisiae* strains and 10 non-*Saccharomyces* yeast strains that utilize proline effectively. Notably, two of these *S. cerevisiae* strains, UCDFST 40-144 and 68-44, utilize proline and produce sufficient alcohol for beer fermentation models. These strains have the potential to create distinctive beer products.

## 2. Materials and Methods

### 2.1. Yeast Strains and Culture Media

A total of 205 yeast strains used in this study are listed in Table S1. All strains were obtained from the Phaff Yeast Culture Collection (University of California, Davis, CA, USA, https://phaffcollection.ucdavis.edu). Three growth media were used in this study. One was a synthetic complete medium with proline (SC+Pro) (2% glucose, 0.5% ammonium sulfate, 0.17% yeast nitrogen base without both amino acid and ammonium sulfate (Difco Laboratories, Detroit, MI, USA), 0.1% proline, 0.04% leucine, 0.008% alanine, 0.008% asparagine, 0.008% aspartic acid, 0.008% glutamine, 0.008% glutamic acid, 0.008% cysteine, 0.008% glycine, 0.008% histidine, 0.008% isoleucine, 0.008% lysine, 0.008% methionine, 0.008% phenylalanine, 0.008% serine, 0.008% threonine, 0.008% tryptophane, 0.008% valine, 0.008% uracil, 0.008%, inositol 0.008% *p*-aminobenzoic acid, and 0.002% adenine). The second was a YMPD medium (1% yeast extract (Difco Laboratories), 1% malt extract (Difco Laboratories), 2% peptone (Difco Laboratories), and 2% glucose). The third was a 10 °P wort (11.4% dried malt extract (Briess Malt & Ingredients, Chilton, WI, USA)).

### 2.2. Measurement of Residual Proline Content

The residual proline content in each supernatant was determined via the isatin colorimetric method [17] or an amino acid analyzer (L-8800, Hitachi, Tokyo, Japan). To measure proline levels using the isatin method, 4-fold diluted samples were mixed with citrate buffer (0.5 M, pH 4.1). A 0.075% (*w*/*v*) solution of isatin dissolved in a 1:2 mixture of acetone and ethanol was added to the mixture. The samples were evaporated to dryness by heating at 99 °C for 10 min. The blue residues of the reaction product of proline and isatin were then dissolved in 66% (*v*/*v*) aqueous acetone, and absorbance at 595 nm of the resulting solution was measured using a microplate reader (VersaMaxTM, Molecular Devices, Sunnyvale, CA, USA). For the determination of proline levels using the amino acid analyzer, samples were diluted more than 2-fold with citrate buffer (0.2 M, pH 2.2). Amino acids in samples were separated by ion-exchange chromatography and were detected by ninhydrin reaction.

### 2.3. Small-Scale Screening of Proline-Utilizing Strains

Yeast cells were grown for 48 h with shaking (30 °C, 200 rpm) in 2 mL of SC+Pro and were then transferred to 2 mL of freshly prepared SC+Pro. After shaking (30 °C, 200 rpm) for 48 h, the supernatant was collected via centrifugation. Proline utilization was evaluated by measuring the residual proline content in each supernatant determined by the isatin colorimetric method.

### 2.4. Large-Scale Screening of Proline-Utilizing Strains

Yeast cells were inoculated into 5 mL of SC+Pro starting from an optical density at 600 nm (OD_600_) of 2.0. After incubation with shaking for the time points indicated in the figure legends, the supernatant was collected via centrifugation. Proline utilization was evaluated by measuring the residual proline content in each supernatant.

### 2.5. Fermentation Test

Yeast cells precultured in YMPD were inoculated into 10 Degrees Plato (°P) wort at the standard ale pitch rate of OD_600_ of 0.1 per °P [18]. The °P value is used to quantify the concentration of extract (mainly sugars derived from the malt but also including other soluble material in the wort) as a percentage by weight (ex. a 10 °P wort has the same density as a water-sucrose solution that contains 10% sucrose by weight). After incubation under static conditions at room temperature for the durations indicated in the figure legends, the supernatants were collected via centrifugation. The clarified supernatant was degassed with an ultrasonic cleaner (B1500A-DTH 1.90 L, VWR International Radnor, Radnor, PA, USA). The alcohol content and °P in each degassed sample were then determined using the Anton Paar Density Meter (DMA 5000 M) and alcolyzer (Alcolyzer Beer M) (Anton Paar USA Inc., Torrance, CA, USA). The proline content was measured with the amino acid analyzer.

### 2.6. Statistical Analysis

Statistical significance was evaluated by using Student’s *t*-test, with a significance level of *p* < 0.05.

## 3. Results and Discussion

**Figure 1:**
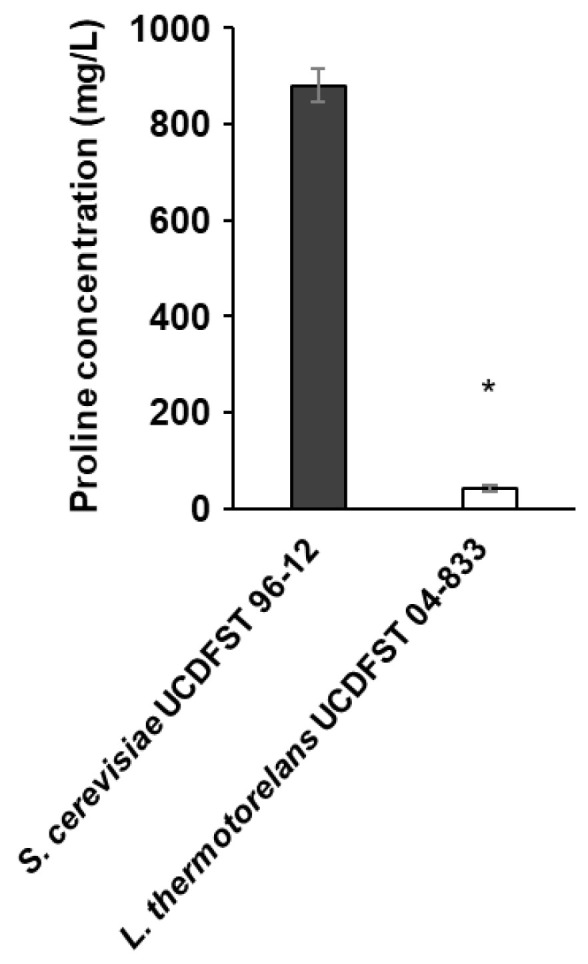
Evaluation of the isatin colorimetric assay to determine proline content. *S. cerevisiae* UCDFST 96-12 and *L. thermotolerans* UCDFST 04-833 were precultured in 2 mL of SC+Pro medium and subsequently transferred to 2 mL of freshly prepared SC+Pro. The residual proline content was determined at 48 h via the isatin colorimetric assay. Data are presented as means ± SD (*n* = 3), and statistical significance was determined using Student’s *t*-test. * *p* < 0.05 vs. *S. cerevisiae* UCDFST 96-12.

Isatin assay was first developed by Boctor [19]. We recently optimized the isatin method for the rapid determination of proline levels [17]. In our study, we demonstrated that this assay is effective for measuring residual proline levels under nitrogen-deficient conditions, such as in a minimal synthetic medium where proline and arginine are the only nitrogen sources. Here, the isatin assay was used to measure proline levels in a beer fermentation model. As wort contains a wide variety and abundance of amino acids [1], we first tested whether the isatin assay could be applied to a nitrogen-rich medium such as a synthetic complete medium. As a model experiment, the American ale yeast *Saccharomyces cerevisiae* UCDFST 96-12 (Wyeast 1056) and the known proline-utilizing yeast *Lachancea thermotolerans* UCDFST 04-833 were cultured in SC+Pro (1000 mg/L). After 72 h, the residual proline was measured using this assay. As shown in Figure 1, after the culture with *S. cerevisiae* UCDFST 96-12, 880 mg/L of proline remained in the medium. This level indicated that this beer yeast strain was unable to utilize proline well. In contrast, *L. thermotolerans* UCDFST 04-833 consumed significantly more proline, leaving only 42 mg/L. The results obtained by the isatin assay were compared with those obtained by an amino acid analyzer, a conventional method for measuring proline (Table 1). *L. thermotolerans* UCDFST 04-833 consumed proline more efficiently than *S. cerevisiae* UCDFST 96-12. The isatin assay showed slightly lower values than those determined by the amino acid analyzer, with a difference of 7–184 mg/L of proline. Despite some unexplained limitations in terms of quantification, the isatin assay was found to be useful even under nitrogen-rich conditions.

**Figure 2:**
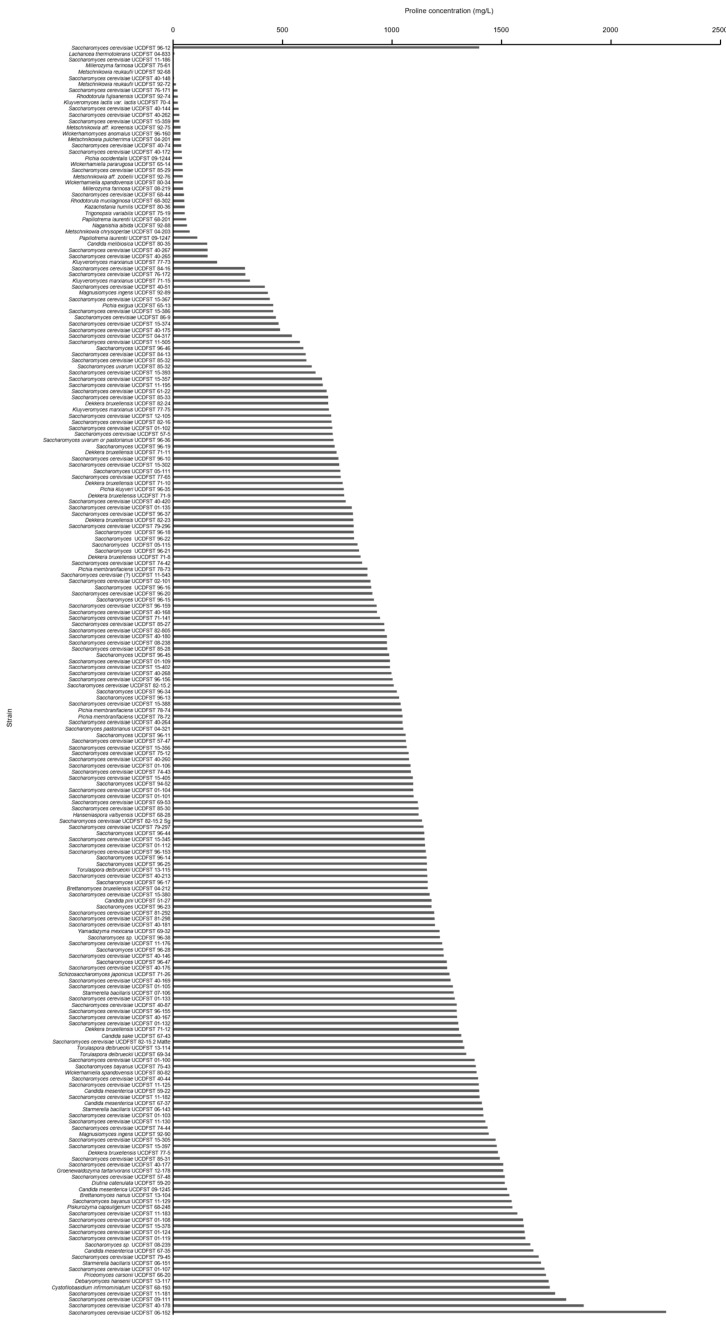
Initial (small-scale) screening of proline-utilizing yeasts. Two hundred and five yeasts isolated from beer/wine fermentations were precultured in 2 mL of SC+Pro medium and subsequently transferred to 2 mL of freshly prepared SC+Pro. The residual proline content was determined at 48 h using the isatin colorimetric assay.

**Figure 3:**
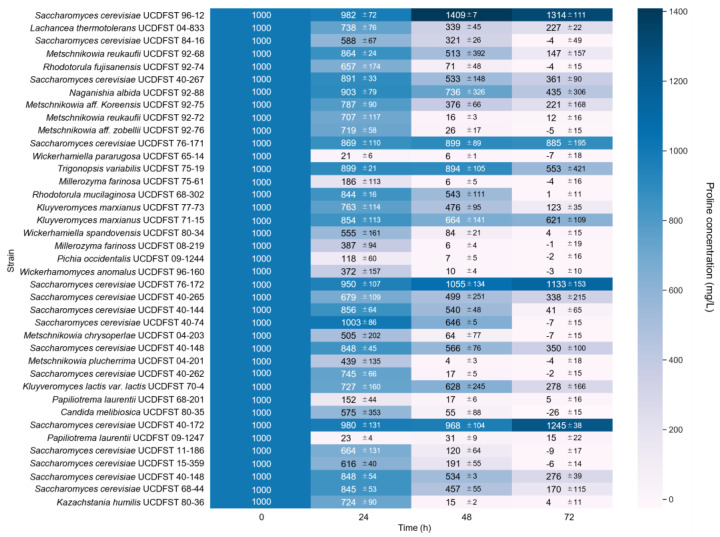
Second (large-scale) screening of proline-utilizing yeasts. Thirty-seven proline-utilizing candidates were inoculated into 5 mL of freshly prepared SC+Pro starting with an OD_600_ of 2.0. The residual proline content was determined at the indicated time points by using the isatin colorimetric assay. The heatmap presents the residual proline level of each strain. White and blue indicate low and high values, respectively. Data are presented as means (*n* = 3).

A variety of species of yeasts from the Phaff Yeast Collection were screened for their ability to utilize proline. First, 205 yeast strains isolated from beer or wine fermentations were selected (Table S1). Table S2 lists the number and percentage of yeasts by genus, with *Saccharomyces* accounting for 69.3% of the total, *Dekkera* for 3.9%, *Candida* for 3.4%, *Pichia* for 2.9%, *Metschnikowia* for 2.9%, and the remaining 22 yeasts for 17.6%. The isatin assay was used to determine the residual proline content in the medium after the cultivation of each strain. As shown in Figure 2, 50.7% of the yeasts, including *S. cerevisiae* UCDFST 96-12, did not consume proline. In some strains, the proline content after cultivation was higher than the initial proline content (1000 mg/L). This result may be due to evaporation of the medium during incubation, resulting in higher proline concentrations [17]. Furthermore, isatin might react with unknown compounds produced by the strains [5]. More importantly, 37 yeast strains (18.0%) reduced proline levels by more than 60%, indicating that these yeasts utilize proline effectively. These 37 yeasts were then subjected to a second screening with a scaled-up culture volume (Figure 3). After the second screening, 31 yeast strains (15.1%) were finally identified as proline-utilizing yeasts, and each reduced proline levels by more than 60%. As shown in Table S3, we analyzed the relationships between genus and proline utilization; within the genus *Saccharomyces*, only 7.7% (11 of 142 strains) were able to utilize proline. These results are consistent with the previous report [16], indicating that *Saccharomyces* species are less efficient in utilizing proline. Interestingly, all yeasts belonging to the genera *Metschnikowia*, *Millerozyma*, *Papiliotrema*, *Rhodotorula*, *Kazachstania*, and *Wickerhamomyces* utilized proline, even though only a few strains were analyzed. These results suggest that proline utilization is more common among non-*Saccharomyces* species rather than in *Saccharomyces*, regardless of nitrogen conditions.

**Figure 4:**
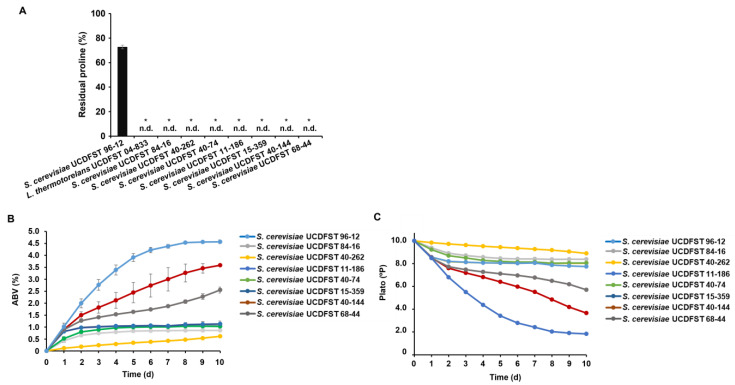
Proline consumption and alcohol production in a beer fermentation model. (**A**) Residual proline levels. *L. thermotolerans* UCDFST 04-833 or *S. cerevisiae* UCDFST 96-12, 84-16, 40-262, 11-186, 40-74, 15-359, 40-144, and 68-44 were inoculated into 10 °P wort, starting with an OD_600_ of 0.1 per °P. The residual proline content was determined at 72 h using the amino acid analyzer. Data are presented as means ± SD (*n* = 3), and statistical significance was determined using Student’s *t*-test. * *p* < 0.05, vs. *S. cerevisiae* UCDFST 96-12. n.d.: not detected (**B**) Alcohol concentrations (alcohol by volume; ABV). *S. cerevisiae* UCDFST 96-12, 84-16, 40-262, 11-186, 40-74, 15-359, 40-144, and 68-44 were inoculated into 10 °P wort starting with an OD_600_ of 0.1 per °P. Alcohol concentrations were determined at the indicated time points by using the alcolyzer. Data are presented as means ± SD (*n* = 2). (**C**) Extracted concentrations (degrees Plato; °P). *S. cerevisiae* UCDFST 96-12, 84-16, 40-262, 11-186, 40-74, 15-359, 40-144, and 68-44 were inoculated into 10 °P wort starting from OD_600_ of 0.1 per °P. The °P value was determined using the alcolyzer at the indicated time points. Data are presented as means ± SD (*n* = 2).

*S. cerevisiae* is well known to produce high concentrations of alcohol and is often used in the production of beer and wine. Therefore, seven proline-utilizing *S. cerevisiae* strains (UCDFST 84-16, 40-262, 11-186, 40-74, 15-359, 40-144, and 68-44) were selected that were able to reduce proline levels by more than 70% (Figure 3). The ability of these strains to utilize proline effectively in a wort-based beer fermentation model was determined. As shown in Figure 4A, *S. cerevisiae* UCDFST 96-12 did not reduce proline levels, whereas *L. thermotolerans* UCDFST 04-833 completely consumed proline in the medium. Intriguingly, all selected *S. cerevisiae* strains utilized proline significantly, as did *L. thermotolerans* UCDFST 04-833. Alcohol concentrations (alcohol by volume; ABV) in beer brewed with these proline-utilizing *S. cerevisiae* strains were determined (Figure 4B). *S. cerevisiae* UCDFST 96-12 produced 4.5% alcohol after 10 days of fermentation. The Plato value decreased to 2.0 °P due to alcohol production (Figure 4C). On the other hand, beer strain *S. cerevisiae* UCDFST 84-16, and wine strains 40-262, 11-186, 40-74, and 15-359 produced only about 1.0% alcohol. The Plato levels in the medium did not change with fermentation, indicating that these strains were unable to utilize maltose in the wort during fermentation. Surprisingly, *S. cerevisiae* UCDFST 40-144 (Tokay wine strain) and 68-44 (Montrachet wine strain) produced 3.6% and 2.6% alcohol, respectively. Plato levels in the medium decreased during the 10 days of fermentation to 3.7 °P for 40-144 and 5.7 °P for 68-44. We determined the consumption of all amino acids by *S. cerevisiae* UCDFST 96-12 or 40-144 and 68-44 (Figure S1). *S. cerevisiae* UCDFST 40-144 and 68-44 consumed significantly higher amounts of all amino acids compared to *S. cerevisiae* UCDFST 96-12. These data suggest that both *S. cerevisiae* UCDFST 40-144 and 68-44 have a higher ability to uptake not only proline but also general amino acids. These data indicate that *S. cerevisiae* UCDFST 40-144 and 68-44 can produce a considerable amount of alcohol by utilizing maltose in the wort during fermentation. Although their fermentation rate was slower than that of ale yeast, these results suggested that *S. cerevisiae* UCDFST 40-144 and 68-44 can utilize proline and produce alcohol even in the beer fermentation model.

In this study, two *S. cerevisiae* strains (UCDFST 40-144 and 68-44) were identified that consume extracellular proline and produce sufficient alcohol in a beer fermentation model. Beer yeasts were initially selected to test proline utilization during beer fermentation. However, the fact that both UCDFST 40-144 and 68-44 strains were originally isolated from wine fermentation conditions indicates that these yeasts may be suitable for both brewing and winemaking (Table S1). Postigo et al. reported that the wine yeast *S. cerevisiae* was able to brew beer with a highly distinct flavor profile (characterized by notes of malt, fruit, hop, banana, and phenolic aromas) not found in beer brewed with commercial beer yeasts [20]. They attributed these characteristics mainly to a high level of ester production. Further analyses including the identification of volatile compounds and sensory evaluation are needed to determine whether these wine yeast strains have the potential to produce distinctive beer flavor profiles.

Previous studies have proposed that proline utilization is negatively regulated by the activation of protein kinase A (PKA) via a mechanism involving glycolysis and the Ras guanine nucleotide exchange factor (GEF) Cdc25 pathway [21]. PKA signaling is mainly activated by the dissociation of the catalytic (Tpk1, 2, 3) and regulatory (Bcy1) subunits of PKA, which is caused by a rapid increase in intracellular cAMP levels. We also reported that basic amino acids such as arginine rapidly activate PKA activity without increasing cAMP levels [22]. The arginine transporter Can1 mediates the activation of PKA using basic amino acids. Specifically, Can1 controls PKA activity by functioning as a transceptor that combines the functions of both transporter and receptor for basic amino acids. These findings indicate that PKA signaling is crucial for inhibiting proline utilization. Therefore, *S. cerevisiae* UCDFST 40-144 and 68-44 may have genotypes associated with PKA signaling. Whole genome sequences of *S. cerevisiae* UCDFST 40-144 and 68-44 would be helpful to understand the detailed mechanism underlying the inhibition of proline utilization via the PKA signaling pathway.

In this study, yeasts belonging to genera *Metschnikowia*, *Millerozyma*, *Papiliotrema*, *Rhodotorula*, *Kazachstania*, and *Wickerhamomyces* utilized proline in the nitrogen-rich medium. Interestingly, *Kazachstania*, *Wickerhamomyces*, and *Metschnikowia* are used for beer fermentation [23,24,25]. *Kazachstania servazzii* produces a sweet taste due to its low utilization of carbon sources but confers cereal, bready, and wort flavors to the beer. Notably, *K*. *servazzii* can produce a high level of phenylethyl acetate. *Wickerhamomyces* contributes to the acidity of beer by converting sugar into lactic acid, resulting in a clean, aromatic, and fruity flavor with notes of pear, apple, and apricot. Non-*Saccharomyces* yeast species are normally used in continuous and mixed fermentations because of their inability to utilize maltose, which can contribute to distinctive flavor profiles. *Metschnikowia*, which is often used in continuous fermentations, produces high levels of isoamyl alcohol and isoamyl acetate, resulting in beer with a well-balanced body and fruity aromas. Unlike these yeasts, *Millerozyma* and *Rhodotorula* have not been used for beer production. *Rhodotorula* has been recognized as a potent source of valuable biomolecules such as carotenoids, lipids, and enzymes [26]. Due to its production of carotenoid pigments and preference for aerobic growth, *Rhodotorula* may not be suitable for beer production. *Millerozyma* has been reported to be involved in the production of acetic acid, phenylethyl alcohol, 3-furaldehyde, and esters in baijiu, a type of Chinese liquor [27]. Since many non-*Saccharomyces* yeasts are used in continuous or mixed fermentations, *Millerozyma* yeast can be used to brew beer with a unique flavor profile.

In conclusion, we reported that the isatin method is suitable for the rapid determination of proline in nitrogen-rich media. We also identified *Saccharomyces* and non-*Saccharomyces* strains that can assimilate proline and produce alcohol in a beer fermentation model. These strains may contribute to the development of distinctive beer products. Furthermore, the Phaff Yeast Culture Collection will continue to have great potential to support industrial applications.

## Figures and Tables

**Table 1:** Proline consumption in *S. cerevisiae* UCDFST 96-12 and *L. thermotorelans* UCDFST 04-833 determined using the isatin method and Amino acid analyzer.

Strain	Time (h)	Proline Concentration (mg/L)	
		by Isatin	by A. A. Analyzer
*S. cerevisiae* UCDFST 96-12	24	990 ± 10.9	1066 ± 39.3
	48	916 ± 22.9	1100 ± 9.2
	72	1003 ± 2.5	1162 ± 5.2
*L. thermotorelans* UCDFST 04-833	24	800 ± 18.4	807 ± 18.0
	48	181 ± 0.0	208 ± 14.6
	72	103 ± 54.2	110 ± 54.9

## Data Availability

Data are contained within the article and supplementary materials.

## Supplementary Materials

The following supporting information can be downloaded at: https://www.mdpi.com/article/10.3390/jof9121137/s1, Figure S1. Consumption profile of all amino acids. *S. cerevisiae* UCDFST 96-12, 40-144, or 68-44 were inoculated into 10 °P wort starting with an OD_600_ of 0.1 per °P. The residual amino acids content was determined at 72 h by the amino acid analyzer. Data are presented as means (n = 3). Asx; Asp and Asn, Glx; Glu and Gln. Table S1: List of strains used in this research. Table S2: Number and ratio of strains sorted by genera. Table S3: Number of strains in selected genera before and after screenings.
