# Supplementary material for: Isolation of Yeast Strains with Higher Proline Uptake and Their Applications to Beer Fermentation
Source: J Fungi (Basel). 2023 Nov 24;9(12):1137. doi: 10.3390/jof9121137 (PMC10744042; doi:10.3390/jof9121137)
Supplement: Supplementary file 1 [file jof-09-01137-s001.zip › jof-2713601-supplementary.pdf]

Supplemental Files

Isolation of yeast strains with higher proline uptake and their applications to beer fermentation

Ryoya Tanahashi [1,2], Akira Nishimura [1,3]*, Minh Nguyen [2], Irnayuli Sitepu [2], Glen Fox [2], Kyria Boundy-Mills [2] and Hiroshi Takagi [1]**

[1]Institute for Research Initiatives, Nara Institute of Science and Technology, 8916-5 Takayama-cho, Ikoma, Nara 630-0192, Japan

[2]Department of Food Science and Technology, University of California Davis, One Shields Ave, Davis, CA, 95616, USA

[3]Division of Biological Science, Graduate School of Science and Technology, Nara Institute of Science and Technology, 8916-5 Takayama-cho, Ikoma, Nara 630-0192, Japan

*Correspondence: nishimura@bs.naist.jp

*Correspondence: hiro@bs.naist.jp

Key words: *Saccharomyces cerevisiae*; non-*Saccharomyces*; proline utilization; yeast culture collection; beer fermentation

This PDF file includes:
Fig. S1, Tables S1-S3

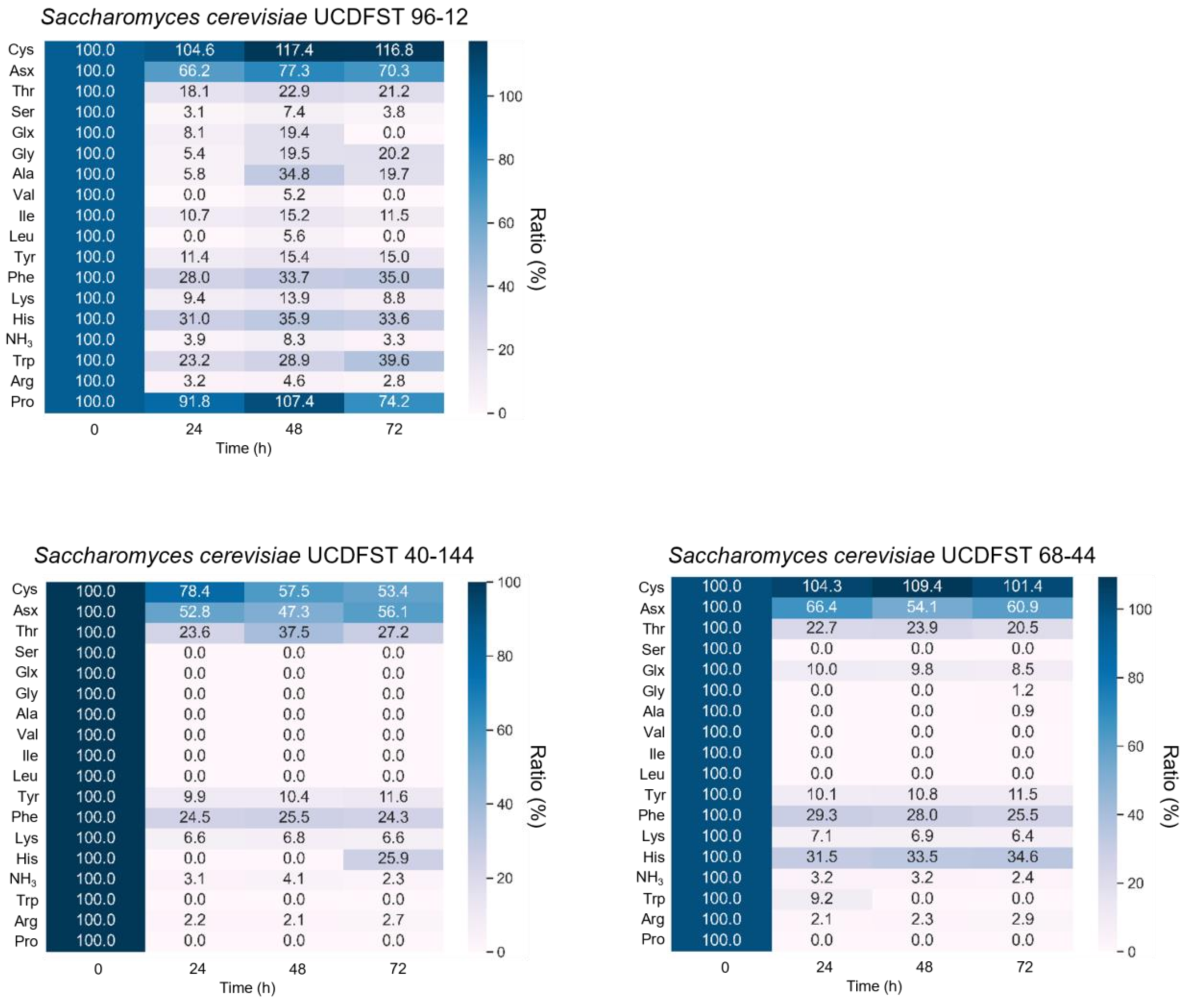

Fig. S1. Consumption profile of all amino acids. *S. cerevisiae* UCDFST 96-12, 40-144, or 68-44 were inoculated into 10 °P wort starting with an OD_{600} of 0.1 per °P. The residual amino acids content was determined at 72 h by the amino acid analyzer. Data are presented as means (n = 3). Asx; Asp and Asn, Glx; Glu and Gln.

Table S1. List of strains used in this research

Name	Geographic origin	Isolate from	Relation: Beer or Wine
Saccharomyces cerevisiae UCDFST 96-12	USA	American ale yeast	Beer (Control)
Lachancea thermotorelance UCDFST 04-833	Wolfskill Experimental Orchard, near Winters, California, USA	Bactrocera oleae (olive fruit fly) adult male	n/a (Control)
Millerozyma farinosa UCDFST 75-61	Poland	Jopen beer	Beer
Metschnikowia reukaufii UCDFST 92-68	Hweihsi Forest, Taiwan	Flowers of Aleurites montana	Beer
Metschnikowia reukaufii UCDFST 92-72	Hweihsi Forest, Taiwan	Flowers of Aleurites montana	Beer
Saccharomyces cerevisiae UCDFST 76-171	San Francisco, California, USA	Isolated from ale starter 12/76, Anchor Steam Beer.	Beer
Rhodotorula fujisanensis UCDFST 92-74	Hsweihsi Forest, Taiwan	Flowers of Aleurites montana	Beer
Metschnikowia aff. koreensis UCDFST 92-75	Hweihsi Forest, Taiwan	Mainly flowers. Tree exudate of Aleurites montana	Beer
Wickerhamomyces anomalus UCDFST 96-160	Davis, California, USA	Bottled beer	Beer
Pichia occidentalis UCDFST 09-1244	Java, Indonesia	Tea-beer fungus	Beer
Wickerhamiella pararugosa UCDFST 65-14	San Francisco, California, USA	Isolated from keg beer, Lucky Lager Co. San Francisco.	Beer
Saccharomyces cerevisiae UCDFST 85-29	Seattle, Washington, USA	Ale yeast	Beer
Metschnikowia aff. zobellii UCDFST 92-76	Hweihsi Forest, Taiwan	Usual in flowers. Tree exudate of Aleurites montana	Beer
Wickerhamiella spandovensis UCDFST 80-34	Germany	German Pilsener beer via Windisch	Beer
Millerozyma farinosa UCDFST 08-219	Danzig, Poland	Jopen beer	Beer
Rhodotorula mucilaginosa UCDFST 68-302	Bremen, Germany	Isolated from pasteurized beer.	Beer
Kazachstania humilis UCDFST 80-36	South Africa	Isolated from Bantu beer in South Africa.	Beer
Trigonopsis variabilis UCDFST 75-19	Germany	Isolated from beer	Beer
Naganishia albida UCDFST 92-88	Hweihsi Forest, Taiwan	Tree exudate of Aleurites montana	Beer
Saccharomyces cerevisiae UCDFST 40-267	USA	Ale yeast	Beer
Saccharomyces cerevisiae UCDFST 40-265	USA	Ale yeast	Beer
Kluyveromyces marxianus UCDFST 77-73	South Africa	Bantu beer by v.d. Walt	Beer
Saccharomyces cerevisiae UCDFST 84-16	UK	Ale yeast	Beer
Saccharomyces cerevisiae UCDFST 76-172	Anchor Steam Beer, San Francisco, California, USA	Isolated from ale starter 12/76	Beer
Kluyveromyces marxianus UCDFST 71-15	South Africa	Isolated from bantu beer in South Africa.	Beer
Magnusiomyces ingens UCDFST 92-89	Hweihsi Forest, Taiwan	Tree exudate of Aleurites montana	Beer
Pichia exigua UCDFST 65-13	San Francisco, California, USA	Isolated from keg beer, Lucky Lager Co. San Francisco.	Beer
Saccharomyces sp. UCDFST 96-46	Unknow	Burton Ale Yeast.	Beer
Saccharomyces cerevisiae UCDFST 84-13	UK	Ale yeast	Beer
Saccharomyces cerevisiae UCDFST 85-32	Hopland, California, USA	Ale yeast	Beer
Saccharomyces uvarum UCDFST 04-316	Japan	BR, Spoiled beer	Beer
Saccharomyces cerevisiae UCDFST 85-33	Unknown	Wheat beer yeast, commercial strain	Beer
Dekkera bruxellensis UCDFST 82-24	Georgia, Atlanta, USA	Isolated from dry ginger ale. Obtained from D. Ahearn.	Beer
Kluyveromyces marxianus UCDFST 77-75	South Africa	Bantu beer	Beer
Saccharomyces cerevisiae UCDFST 12-105	Unknow	British Ale Yeast, WLP005	Beer
Saccharomyces cerevisiae UCDFST 82-16	Thousand Oaks, California, USA	Lager beer yeast obtained from Thousand Oaks Brewery.	Beer
Saccharomyces cerevisiae UCDFST 57-5	England	Isolated from super-attenuated beer	Beer
Saccharomyces uvarum or *pastorianus* UCDFST 96-36	California, USA	Lager strain; Isolated by B.M. Brown(1951); Whitbread strain; Burton Ale Yeast;	Beer
Saccharomyces pastorianus UCDFST 96-19	USA	American Lager Yeast.	Beer
Dekkera bruxellensis UCDFST 71-11	Unknown	Isolated from a bottle of spoiled beer.	Beer
Saccharomyces cerevisiae UCDFST 96-10	Germany	German Ale Yeast (prob. cerevisiae).	Beer
Saccharomyces cerevisiae UCDFST 05-111	Kalamazoo, Michigan USA	Oberon Ale Yeast (wheat ale)	Beer
Saccharomyces cerevisiae UCDFST 77-65	Sonoma, California, USA	Ale yeast	Beer
Dekkera bruxellensis UCDFST 71-10	Unknown	Isolated from a bottle of spoiled beer.	Beer
Pichia kluyveri UCDFST 96-35	Unknown	"Ale Strain; Isolated by: B.M. Brown(1951); Whitbread strain, MUCL 30938;"	Beer
Dekkera bruxellensis UCDFST 71-9	Unknown	Isolated from a keg of spoiled beer.	Beer
Saccharomyces cerevisiae UCDFST 40-420	Copenhagen, Denmark	ale; brewery top yeast originally from Kongens Bryghus in Copenhagen.	Beer
Saccharomyces bayanus UCDFST 01-135	Italy	Isolated from turbid beer	Beer
Saccharomyces bayanus UCDFST 96-37	England	Lager strain.	Beer
Dekkera bruxellensis UCDFST 82-23	South Africa	Isolated from tea-beer in South Africa by J.P. van der Walt.	Beer
Saccharomyces pastorianus UCDFST 96-18	Unkown	Pilsen Lager Yeast.	Beer
Saccharomyces pastorianus UCDFST 96-22	Unkown	Bohemian Lager Yeast.	Beer
Saccharomyces aff. cerevisiae UCDFST 05-115	New Belgian Trippel Ale, Ft Collins, Colorado, USA	New Belgian Trippel Ale, Ft Collins, Colorado.	Beer
Saccharomyces pastorianus UCDFST 96-21	California, USA	California Lager Yeast.	Beer
Dekkera bruxellensis UCDFST 71-8	Unknown	Isolated from a bottle of spoiled beer.	Beer
Saccharomyces cerevisiae UCDFST 11-543	Unknown	Brewing yeast, lager type, Augustiner Type	Beer
Saccharomyces cerevisiae UCDFST 02-101	Ukiah, California, USA	Blue Heron Pale ale, sediment at bottom of bottle	Beer
Saccharomyces cerevisiae UCDFST 96-16	Scotland	Scottish Ale Yeast.	Beer
Saccharomyces cerevisiae UCDFST 96-20	Denmark	Danish Lager Yeast.	Beer
Saccharomyce cerevisiae var. diastaticus UCDFST 96-15	Europe	European Ale Yeast.	Beer
Saccharomyces cerevisiae UCDFST 96-159	Stockton, California, USA	Barley wine beer	Beer
Saccharomyces cerevisiae UCDFST 71-141	Ireland	Isolated from Guinness Stout beer in Ireland; via Dr. Ballou at UCB 10/71.(different isolate from 71-140)	Beer
Saccharomyces cerevisiae UCDFST 85-27	Victoria, British Columbia, Canada	Ale yeast	Beer
Saccharomyces cerevisiae UCDFST 82-805	Unknown	Tesguino ("beer" made from corn)	Beer
Saccharomyces cerevisiae UCDFST 08-238	Saint Lois, Missouri, USA	Ale yeast strain 1044, primarily used for ales at Anheuser Busch. Strain also called "Bud American Ale".	Beer
Saccharomyces cerevisiae UCDFST 85-28	Victoria, British Columbia, Canada	Ale yeast	Beer
Saccharomyces sp. UCDFST 96-45	Unknown	Anchor Ale Yeast.	Beer
Saccharomyces cerevisiae UCDFST 40-268	USA	Ale yeast	Beer
Saccharomyces cerevisiae UCDFST 96-156	Stockton, California, USA	Red beer yeast slurry	Beer
Saccharomyces cerevisiae UCDFST 82-15.2	Unknown	Lager beer yeast obtained from Maynard Dimond.	Beer
Saccharomyces cerevisiae UCDFST 96-34	Belgium	Belgian White Beer Yeast.	Beer
Saccharomyces cerevisiae UCDFST 96-13	Ireland	Irish Ale Yeast.	Beer
Saccharomyces cerevisiae UCDFST 15-388	Missouri, USA	Isolated from beer	Beer
Saccharomyces cerevisiae UCDFST 40-264	USA	Ale yeast	Beer
Saccharomyces pastorianus UCDFST 04-321	Copenhagen, Denmark	Beer, Brewer's yeast	Beer
Saccharomyces cerevisiae UCDFST 96-11	London, England	London Ale Yeast	Beer
Saccharomyces cerevisiae UCDFST 57-47	Azusa, California, USA	Lucky Lager brewing strain	Beer
Saccharomyces cerevisiae UCDFST 94-52	Unknown	Lager yeast	Beer
Saccharomyces cerevisiae UCDFST 69-53	Nutfield, UK	brewing yeast, ale. Used commercially in APV tower fermenters.	Beer
Saccharomyces cerevisiae UCDFST 85-30	Portland,Oregon, USA	Ale yeast	Beer
Hanseniaspora valbyensis UCDFST 68-28	Hungary	From beer (H. de Graaf)	Beer
Saccharomyces cerevisiae UCDFST 82-15.2 Sg	Unknown	Lager beer yeast obtained from Maynard Dimond.	Beer
Saccharomyces sp. UCDFST 96-44	Unknown	Anchor Lager Yeast.	Beer
Saccharomyces cerevisiae UCDFST 96-14	England	British Ale Yeast.	Beer
Saccharomyces pastorianus UCDFST 96-25	Unknown	Munich Lager Yeast.	Beer
Saccharomyces cerevisiae UCDFST 96-17	London, England	Special London Ale Yeast.	Beer
Brettanomyces bruxellensis UCDFST 04-212	Brussels, Belgium	Lambic beer	Beer
Saccharomyces cerevisiae UCDFST 15-380	West Africa	Isolated from beer	Beer
Candida pini UCDFST 51-27	Sweden	Isolated from heartwood of live pines , by Lagerberg	Beer
Saccharomyces pastorianus UCDFST 96-23	Unknown	Bavarian Lager Yeast.	Beer
Saccharomyces sp. UCDFST 96-38	Unknown	Ale Strain.	Beer
Saccharomyces UCDFST 96-47	Unknown	St Stan's Ale Yeast.	Beer
Dekkera bruxellensis UCDFST 71-12	Unknown	Isolated from a bottle of spoiled beer.	Beer
Candida sake UCDFST 67-43	Belgium	Isolated by H. Kufferath from lambic beer, Belgium.	Beer
Saccharomyces cerevisiae UCDFST 82-15.2 Matte	Unknown	Lager beer yeast obtained from Maynard Dimond.	Beer
Torulaspora delbrueckii UCDFST 69-34	Unknown	From spoiled ale.	Beer
Wickerhamiella spandovensis UCDFST 80-82	Germany	isolated from German Pilsener beer by W. Henninger	Beer
Candida mesenterica UCDFST 59-22	Lucky Lager Brewery, San Francisco, California, USA	Isolated from fermenting beer wort	Beer
Candida mesenterica UCDFST 67-37	Holland	Isolated from beer conduit tubes, Holland, by R. Vermeulen.	Beer
Magnusiomyces ingens UCDFST 92-90	Hweihsi Forest, Taiwan	Tree exudate of Aleurites montana	Beer
Saccharomyces cerevisiae UCDFST 85-31	Portland, Oregon, USA	Ale yeast	Beer
Saccharomyces cerevisiae UCDFST 57-48	Azusa, California, USA	Lucky Lager brewing strain	Beer
Diutina catenulata UCDFST 59-20	Lucky Lager Brewery, San Francisco, California, USA	Isolated from fermenting beer wort	Beer
Candida mesenterica UCDFST 09-1245	Germany	Beer pipes in brewery	Beer
Brettanomyces nanus UCDFST 13-104	Kalmar, Sweden	Bottled beer	Beer
Saccharomyces sp. UCDFST 08-239	Saint Lois, Missouri, USA	Strain W205, a hefeweizen strain. "W" may stand for "Weinhenstephan", at theUniversity of Munich, brewing program. Used at Anheuser Busch to make wheat beers.	Beer
Candida mesenterica UCDFST 67-35	Germany	Isolated from beer conduit tubes, Germany by H. schnegg.	Beer
Cystofilobasidium infirmominiatum UCDFST 68-193	Stockholm, Sweden	Isolated from a beer bottle washer.	Beer
Saccharomyces cerevisiae UCDFST 11-186	South Africa	Wine	Wine
Saccharomyces cerevisiae UCDFST 40-148	Frei Bros. winery, California, USA	Naturally fermenting vat of wine	Wine
Kluyveromyces lactis var. lactis UCDFST 70-4	South Africa	Isolated in South Africa from winery equipment by v.d. Walt.	Wine
Saccharomyces cerevisiae UCDFST 40-144	Unknown	Wine	Wine
Saccharomyces cerevisiae UCDFST 40-262	State of San Paulo ,Brazil	Rosati Wine	Wine
Saccharomyces cerevisiae UCDFST 15-359	South Africa	Isolated from wine	Wine
Metschnikowia pulcherrima UCDFST 04-201	Unknown	Isolated from barrel fermentation (wine?)	Wine
Saccharomyces cerevisiae UCDFST 40-74	California, USA	Wine	Wine
Saccharomyces cerevisiae UCDFST 40-172	Unknown	Sherry wine strain.	Wine
Saccharomyces cerevisiae UCDFST 68-44	France	Montrachet, Enology strain.	Wine
Papiliotrema laurentii UCDFST 68-201	Belgian Congo	Isolated from "malafou" (palm wine).	Wine
Metschnikowia chrysoperlae UCDFST 04-203	USA, California, Napa	Isolated from barrel fermentation (wine?)	Wine
Papiliotrema laurentii UCDFST 09-1247	Congo	Palm wine	Wine
Candida melibiosica UCDFST 80-35	Japan	Isolated from wine in Japan by Goto.	Wine
Saccharomyces cerevisiae UCDFST 40-51	France	Burgundy wine yeast	Wine
Saccharomyces cerevisiae UCDFST 15-367	Japan	Isolated from wine	Wine
Saccharomyces cerevisiae UCDFST 15-386	Africa	Isolated from wine	Wine
Saccharomyces cerevisiae UCDFST 86-9	Probably Germany	Gesenheim wine strain	Wine
Saccharomyces cerevisiae UCDFST 15-374	France	Isolated from wine	Wine
Saccharomyces cerevisiae UCDFST 40-175	Unknown	Zinfandel wine strains.	Wine
Saccharomyces cerevisiae UCDFST 04-317	Switzerland	Wine	Wine
Saccharomyces cerevisiae UCDFST 11-505	France	Wine	Wine
Saccharomyces cerevisiae UCDFST 15-393	Unknown	Isolated from wine	Wine
Saccharomyces cerevisiae UCDFST 15-357	Italy	Isolated from wine	Wine
Saccharomyces cerevisiae UCDFST 11-195	Japan	Wine	Wine
Saccharomyces cerevisiae UCDFST 61-22	Ivory Coast, South Africa	Isolated from palm wine Type strain of Saccharomyces chevalieri.	Wine
Saccharomyces cerevisiae UCDFST 01-102	Unknown	Commercial wine strain K-1, UCD VE # 960. Obtained by VE from Scott Labs, Lalvin, 1993.	Wine
Saccharomyces cerevisiae UCDFST 15-302	Nigeria	Isolated from wine	Wine
Saccharomyces cerevisiae UCDFST 79-296	Unknown	Wine yeast "Ausmanns hausen" obtained from wine consultant L. Wara	Wine
Saccharomyces cerevisiae UCDFST 74-42	USA, California	Isolated in Phaff's lab from a sample of Chenin Blanc wine from Chappellet Vineyards as a majority isolates. 6-74.	Wine
Pichia membranifaciens UCDFST 78-73	Unknown	isolated by Paulette from bottled wine	Wine
Saccharomyces cerevisiae UCDFST 40-168	Unknown, possibly Spain	Wine	Wine
Saccharomyces cerevisiae UCDFST 40-180	Unknown	Riesling Wine strain	Wine
Saccharomyces cerevisiae UCDFST 01-109	Unknown	Commercial wine strain Montrachet	Wine
Saccharomyces cerevisiae UCDFST 15-402	Nigeria	Isolated from wine	Wine
Pichia membranifaciens UCDFST 78-74	Unknown	Isolated by Paulette from bottled wine	Wine
Pichia membranifaciens UCDFST 78-72	Unknown	Isolated by Paulette from bottled wine	Wine
Saccharomyces cerevisiae UCDFST 15-356	Italy	Isolated from wine	Wine
Saccharomyces cerevisiae UCDFST 75-12	Spain	Wine strain Flor, Universal Food Corp.	Wine
Saccharomyces cerevisiae UCDFST 40-260	Martinez, California, USA	Di Gardi Sweet wine	Wine
Saccharomyces cerevisiae UCDFST 01-106		Commercial wine strain Premier Cuvee, Red Star, UCD VE # 905.	Wine
Saccharomyces bayanus UCDFST 74-43	USA, California	Isolated in Dr. Phaff's lab from a sample of Riesling wine from Chappellet Vineyards as the only occurring yeast. 6-74.	Wine
Saccharomyces cerevisiae UCDFST 15-405	Slovenia	Isolated from wine	Wine
Saccharomyces cerevisiae UCDFST 01-104	Unknown	Commercial wine strain 71 B, UCD VE # 959.	Wine
Saccharomyces cerevisiae UCDFST 01-101		Commercial wine strain Souche, ICV D47, UCD VE # 963. Obtained by VE from Vinquiry, Enoferm, 1993.	Wine
Saccharomyces cerevisiae UCDFST 79-297	Unknown	Wine yeast "Champagne" obtained from wine consultant L. Wara	Wine
Saccharomyces cerevisiae UCDFST 15-345	Africa	Isolated from wine	Wine
Saccharomyces cerevisiae UCDFST 01-112	France, Bouzy	Wine strain Bouzy	Wine
Saccharomyces cerevisiae UCDFST 96-153	Stockton, California, USA	Barley wine yeast slurry	Wine
Torulaspora delbrueckii UCDFST 13-115	Chateau Luchey-Halde, Bordeaux, France	Grape wine (Merlot), 2007	Wine
Saccharomyces cerevisiae UCDFST 40-213	Minas Gerais, Brazil	Strain "Rimac" isolated from Rimac Wine	Wine
Saccharomyces cerevisiae UCDFST 81-292	Unknown	Champagne wine	Wine
Saccharomyces cerevisiae UCDFST 79-298	Probably Germany	Wine yeast "Trockenbeeren auslesen"	Wine
Saccharomyces cerevisiae UCDFST 40-181	Unknown	Winnigen Wine strain	Wine
Yamadazyma mexicana UCDFST 69-32	Italy	Isolated in Italy from wine must by Florenzano in 1953.	Wine
Saccharomyces cerevisiae UCDFST 11-176	California, USA	Wine	Wine
Saccharomyces sp. UCDFST 96-28	Unknown	Red Wine Yeast, Wyeast#3028	Wine
Saccharomyces cerevisiae UCDFST 40-146	Champagne, France	Wine strain Champagne Ay C.	Wine
Saccharomyces cerevisiae UCDFST 40-176	Unknown	Waldenburg wine strain.	Wine

Schizosaccharomyces japonicus UCDFST 71-26	Japan	Strawberry wine	Wine
Saccharomyces cerevisiae UCDFST 40-169	Unknown	Steinberg wine strain.	Wine
Saccharomyces cerevisiae UCDFST 01-105	Unknown	Commercial wine strain M2, UCD VE # 906.	Wine
Starmerella bacillaris UCDFST 07-106	Bordeaux, France	Wine fermentation	Wine
Saccharomyces cerevisiae UCDFST 01-133	Unknown	Simi white (wine?) strain	Wine
Saccharomyces cerevisiae UCDFST 40-87	France, Sauterne	Sauterne wine	Wine
Saccharomyces cerevisiae UCDFST 96-155	Stockton, California, USA	Barley wine yeast slurry	Wine
Saccharomyces cerevisiae UCDFST 40-167	Unknown, possibly Germany	Geisenheim Wine Strain.	Wine
Saccharomyces cerevisiae UCDFST 01-132	France	Wine strain Prise de Mousse Champagne, Pasteur Institute	Wine
Torulaspora delbrueckii UCDFST 13-114	Bordeaux, France	Grape wine (Merlot), 2007	Wine
Saccharomyces cerevisiae UCDFST 01-100		Commercial wine strain Cote des Blancs, UCD VE # 2031.	Wine
Saccharomyces bayanus UCDFST 75-43	California, USA	Isolated from commercial Zinfandel wine	Wine
Saccharomyces cerevisiae UCDFST 40-44	Unknown	Champagne wine yeast.	Wine
Saccharomyces cerevisiae UCDFST 11-125	Nigeria	Palm wine	Wine
Saccharomyces cerevisiae UCDFST 11-182	Italy	Wine	Wine
Starmerella bacillaris UCDFST 06-143	Napa, California, USA	Sweet wine fermentation (Dolce), semillon grape juice	Wine
Saccharomyces cerevisiae UCDFST 01-103	Italy	Unknown	Wine
Saccharomyces bayanus UCDFST 11-130	Tinukari, North Kolaka, Sulawesi, Indonesia	Tree on trail. Scarab larva 017. Gut dissection.	Wine
Saccharomyces cerevisiae UCDFST 74-44	Chappellet Vineyards, California, USA	Isolated in Dr. Phaff's lab from Chenin Blanc wine as minority organism. 6/74	Wine
Saccharomyces cerevisiae UCDFST 15-305	Slovenia	Isolated from wine	Wine
Saccharomyces cerevisiae UCDFST 15-397	Europe	Isolated from wine	Wine
Dekkera bruxellensis UCDFST 77-5	Unknown	Unknown	Wine
Saccharomyces cerevisiae UCDFST 40-177	Europe	European winery, Bordeaux wine strain.	Wine
Groenewaldozyma tartarivorans UCDFST 12-178	Torres Vedras, Runa, Portugal	Dried lees of wine	Wine
Saccharomyces bayanus UCDFST 11-129	North Carolina, USA	Wine	Wine
Piskurozyma capsuligenum UCDFST 68-248	South Africa	Isolated from wine cellar material v.d. Walt.	Wine
Saccharomyces cerevisiae UCDFST 11-183	Italy	Wine	Wine
Saccharomyces cerevisiae UCDFST 01-108	Unknown	Commercial wine strain French White	Wine
Saccharomyces cerevisiae UCDFST 15-378	France	Isolated from wine	Wine
Saccharomyces cerevisiae UCDFST 01-124	Hungary, Tokay region	Wine strain Tokay	Wine
Saccharomyces cerevisiae UCDFST 01-119	Unknown	Wine strain Muscatel	Wine
Saccharomyces cerevisiae UCDFST 79-45	Spain	Isolated from white wine by J. Santa Maria	Wine
Starmerella bacillaris UCDFST 06-151	Tokaj wine region, Hungary	Botrytized wine	Wine
Saccharomyces cerevisiae UCDFST 01-107	Unknown	Commercial wine strain Pasteur Champagne, Red Star	Wine
Priceomyces carsonii UCDFST 66-20	Germany	Bottle of spoiled wine	Wine
Debaryomyces hansenii UCDFST 13-117	Bordeaux, France	Grape/wine	Wine
Saccharomyces cerevisiae UCDFST 11-181	Italy	Wine	Wine
Saccharomyces cerevisiae UCDFST 09-111	Unknown	wine	Wine
Saccharomyces cerevisiae UCDFST 40-178	Unknown	Muscatel wine strain.	Wine
Saccharomyces cerevisiae UCDFST 06-152	Tokaj wine region, Hungary	Wine	Wine

Table S2. Number and ratio of strains sorted by genera.

Genus	Number	Ratio (%)
Saccharomyces	142	69.3
Dekkera	8	3.9
Candida	7	3.4
Pichia	6	2.9
Metschnikowia	6	2.9
Kluyveromyces	4	2.0
Wickerhamiella	3	1.5
Torulaspora	3	1.5
Starmerella	3	1.5
Rhodotorula	2	1.0
Papiliotrema	2	1.0
Millerozyma	2	1.0
Magnusiomyces	2	1.0
Brettanomyces	2	1.0
Yamadazyma	1	0.5
Wickerhamomyces	1	0.5
Trigonopsis	1	0.5
Schizosaccharomyces	1	0.5
Priceomyces	1	0.5
Piskurozyma	1	0.5
Naganishia	1	0.5
Kazachstania	1	0.5
Hanseniaspora	1	0.5
Groenewaldozyma	1	0.5
Diutina	1	0.5
Debaryomyces	1	0.5
Cystofilobasidium	1	0.5
Total	205	100.0

Table S3. Number of strains in selected genera before and after screenings.

Genus	Number of strains		Ratio (%)
	Subjected	Obtained	
Saccharomyces	142	11	7.7
Candida	7	1	14.3
Metschnikowia	6	6	100.0
Pichia	6	1	16.7
Kluyveromyces	4	2	50.0
Wickerhamiella	3	2	66.7
Millerozyma	2	2	100.0
Papiliotrema	2	2	100.0
Rhodotorula	2	2	100.0
Kazachstania	1	1	100.0
Wickerhamomyces	1	1	100.0
